# A comparison of intra-operative diagnosis to histopathological diagnosis of acute appendicitis in paediatric and adult cohorts: an analysis of over 1000 patients

**DOI:** 10.1007/s11845-021-02770-5

**Published:** 2021-09-13

**Authors:** Johnathon Harris, Christina A. Fleming, Paul N. Stassen, Daniel Mullen, Helen Mohan, James Foley, Anna Heeney, Emmeline Nugent, Karl Schmidt, Ken Mealy

**Affiliations:** Department of General Surgery, Wexford General Hospital, Wexford, Ireland

**Keywords:** Acute appendicitis, Histopathology, Intra-operative diagnosis, Pathology

## Abstract

**Background:**

Appendicitis is a common general surgical emergency. The role of removing a normal appendix is debated. However, this relies on accurate intra-operative diagnosis of a normal appendix by the operating surgeon. This study aimed to compare surgeon’s intra-operative assessment to final histological result acute appendicitis in paediatric and adult patients.

**Methods:**

All patients who underwent appendicectomy over a 14-year period in a general surgical department were identified using the prospective Lothian Surgical Audit system and pathology reports retrieved to identify final histological diagnosis. Open appendicectomy was selected to examine, as the routine practise at our institution is to remove a normal appendix at open appendicectomy.

**Results:**

A total of 1035 open appendicectomies were performed for clinically suspected appendicitis. Sensitivity of intra-operative diagnosis of appendicitis with operating surgeon was high at 95.13% with no difference between trainee and consultant surgeon or between adult and paediatric cases. Specificity of intra-operative diagnosis was lower in the paediatric group (32.58%) than in the adult group (40.58%). Women had a higher rate of negative appendicectomy than men.

**Conclusion:**

The results of this study highlight some discordance between histological evidence of acute appendicitis and intra-operative impression. Therefore other clinical variables and not just macroscopic appearance alone should be used when deciding to perform appendicectomy.

## Introduction

Appendicitis is a common general surgical emergency. Increasingly, a laparoscopic approach is used [1]. Removal of a normal appendix found intra-operatively has been a controversial topic with studies arguing for both proceeding with appendicectomy when a ‘normal’ appendix is found versus leaving the normal appendix [2]. In the traditional open approach, an appendix is typically always removed, even when found to be macroscopically normal. However, removal of a normal appendix is no longer routinely advocated, especially using a laparoscopic approach. This is supported by studies showing low false negative rates and others showing similarities in morbidity regardless of acute appendicitis being present or not [3–5]. However, this practice relies on accurate diagnosis of a normal appendix by the operating surgeon, for which some studies report as being challenging [6, 7]. Current evidence demonstrates that there can be significant discordance between intra-operative and histological diagnosis of acute appendicitis, particularly in female patients [7–9]. Philips et al. demonstrated a high false negative rate on laparoscopic diagnosis of acute appendicitis with almost one third of macroscopically normal appendixes revealing evidence of appendicitis on histology, whilst at open appendicectomy reported only one intra-operative false negative out of 73 patients [6].

This study aimed to compare surgeon intra-operative and pathologist histological assessment of appendicitis following open appendicectomy in paediatric and adult patients in Wexford General Hospital over a 14-year period and to identify factors associated with discordant diagnosis.

## Methods

### Study setting and patient selection

Data on all general surgery patients undergoing operative intervention are prospectively recorded in the Lothian audit system at our institution and pathological reports are centrally maintained. All patients, including paediatric cases, who underwent an open appendicectomy over a 14-year period were assessed for inclusion. Currently it is routine practice in our institution to perform an appendicectomy when a normal appearing appendix is found at the time of open appendicectomy and at laparoscopic appendicectomy the decision to remove is at the discretion of the operating surgeon. Clinical and demographic features were recorded including patient age and gender, the primary operating surgeon (consultant or trainee), the intra-operative diagnosis and histopathological diagnosis classified as uncomplicated appendicitis and presence or absence of the following: perforation, abscess, tumour or a normal appendix.

### Operative approach and histopathological assessment

For patients included in this study all procedures were performed using a standard open appendicectomy technique as follows: surgical antibiotic prophylaxis was administered as a single dose of broad-spectrum antibiotics (usually co-amoxiclav 1.2 g for patients weighing > 30 kg). For patients with a penicillin allergy, cefuroxime 1.5 g was administered. Where the trainee was the primary operator, the supervising consultant was always present in theatre either scrubbed or unscrubbed, but the trainee’s individual assessment of the appendix for presence/absence of appendicitis was recorded. Following appendicectomy all surgical specimens were placed in formalin for transfer to the histopathology department. Appendix specimens were examined in three sections: (1) a cut section from the base of the appendix, (2) a longitudinal section from the middle part of the appendix and (3) a transection of the tip for tip assessment. A macroscopic diagnosis of acute appendicitis was made intra-operatively if any of the following criteria from the Laparoscopic APPendicitis (LAPP) score were met (in the opinion of the primary surgeon) [10]: thickened appendix or mesoappendix; injected serosal vessels; presence of inflammatory adhesions, fibrin or purulent fluid or pus; and evidence of gangrene, necrosis or perforation. A histopathological diagnosis of acute appendicitis was made if any of the following criteria were identified: presence of cells of acute inflammation or engorged blood vessels, fibrinous debris within the wall or signs of gangrenous change or perforation. Presence of lymphocytes in isolation was not diagnostic as this can occur in the setting of systemic viral disease, e.g. mesenteric adenitis.

### Statistical analysis

Statistical analysis was performed using SPSS, version 21. Univariable analysis was performed using Fisher’s exact test and *χ*^2^ where appropriate and *p*-values were considered significant if < 0.05. Sensitivity, specificity, positive predictive values (PPV) and negative predictive values (NPV) were calculated using Bayesian statistics. Multivariable analysis was performed using the predictive power of histological diagnosis of acute appendicitis based on intra-operative findings.

## Results

### Demographic and clinical characteristics

A total of 1035 patients were suitable for inclusion. Patient characteristics are summarised in Table 1. Median age at presentation was 17 years (interquartile range [IQR] 17). Of the patients, 62.6% were male (*n* = 648). Subgroup classification identified 51.1% (*n* = 528) as paediatric cases. A median age of 11.7 years (IQR 3.3) was observed in the paediatric group and 33.2 years (IQR 13.8) in the adult group. The majority of cases, 79.2% (*n* = 820), were performed by a surgical trainee as the primary operator. Similar sex (*p* = 0.06) and primary operator (*p* = 0.148) characteristics were observed for both paediatric and adult groups.

**Table 1:** Demographic and clinicopathological characteristics

		**Total** (*n* = 1035) (%)	**Paediatric cases** (*n* = 528, 51.1%) (%)	**Adult cases** (*n* = 507, 48.9%) (%)	***p*-value**
**Age**	Median	17	11.7	33.2	< 0.001
	IQR	17	3.3	13.8	
**Sex**	Male	648 (62.6)	318 (60.2)	330 (65.1)	0.06 (Fisher’s exact test)
	Female	387 (37.4)	210 (39.8)	177 (34.9)	
**Primary operator**	Consultant	215 (20.8)	117 (22.2)	98 (19.3)	0.148 (Fisher’s exact test)
	Trainee	820 (79.2)	411 (77.8)	409 (80.7)	
**Intra-operative diagnosis**	Appendicitis	802 (77.5)	410 (77.7)	392 (77.3)	0.834 (x^2^)
	Perforation	115 (11.1)	60 (11.4)	55 (10.8)	
	Abscess	16 (1.5)	7 (1.3)	9 (1.8)	
	Tumour	1 (0.1)	0	1 (0.2)	
	Normal Appendix	101 (9.8)	51 (9.6)	50 (9.9)	
**Histopathological Diagnosis**	Appendicitis	863 (83.4)	434 (82.2)	429 (84.6)	0.189 (*χ*^2^)
	Perforation	10 (1.0)	5 (0.9)	5 (1.0)	
	Abscess	1 (0.1)	0	1 (0.2)	
	Tumour	3 (0.3)	0	3 (0.6)	
	Normal appendix	158 (15.3)	89 (16.9)	69 (13.6)	

Comparing adult and paediatric cases, statistical significance observed as *p* < 0.05

*IQR* interquartile range

### Intra-operative and histopathological findings

As outlined in Table 1, from all patients included in this study, the primary operator made an intra-operative diagnosis of appendicitis in 77.5% (*n* = 802), a perforation in 11.1% (*n* = 115), an abscess in 1.5% (*n* = 16), a tumour in 0.1% (*n* = 1) and a normal appendix in 9.8% (*n* = 101). The variation of diagnosis between paediatric cases and adult cases was minimal and not significant (*p* = 0.834). On the histopathological diagnosis of all cases, appendicitis was seen in 83.4% (*n* = 863), a perforation in 1% (*n* = 10), an abscess in 0.1% (*n* = 1), tumours in 0.3% (*n* = 3) and a normal appendix in 15.3% (*n* = 158). The variation of diagnosis between paediatric cases and adult cases was less similar under histopathological analysis compared to intra-operative diagnosis, but still not significant (*p* = 0.189). A greater proportion of paediatric cases had a normal appendix (16.9%, *n* = 89) versus adult cases (13.6%, *n* = 69). Additionally, tumours were identified in three adult specimens on histopathology versus no tumours in the paediatric cohort.

Of the three tumours confirmed on pathological analysis, two were invasive tumours and one was a dysplastic tumour with the primary operator as a trainee in two cases and consultant in one case. All three cases were male patients: two were aged 59 and one aged 63. The dysplastic tumour (villous adenoma with high-grade dysplasia) was diagnosed in a normal appearing appendix and an intra-operative impression of acute appendicitis was made for the other two tumours (with abscess formation for one tumour, a low-grade appendiceal mucinous neoplasm). The second tumour was a mucin producing goblet cell carcinoma and considered to be acute appendicitis intra-operatively. None of the three tumours were identified intra-operatively. In one case, a tumour was suspected intra-operatively and the final histology returned as acute suppurative appendicitis.

### Factors associated with concordance intra-operative and histopathological diagnosis

The sensitivity, specificity, positive (PPV) and negative (NPV) predictive values were calculated based on the diagnostic capability of intra-operative findings (Table 2). This included appendicitis and normal appendix cases confirmed on histopathology (*n* = 1021). The overall sensitivity of intra-operative findings was high at 95.13% (confidence interval [CI] 93.48–96.47), with similar sensitivities between the paediatric and adult groups. The specificity of intra-operative findings was greater in the adult group at 40.58% (CI 28.91–53.08) compared to the paediatric group at 32.58% (CI 32.02–43.34). Both the PPV and NPV of intra-operative diagnosis are greater in the adult group (90.89% and 58.33%, respectively) compared to the paediatric group (87.28% and 56.86%, respectively).

**Table 2:** Diagnostic accuracy of intra-operative findings (*n* = 1021, positive and negative on histological examination only)

	**Total** % (95% CI)	**Paediatric** % (95% CI)	**Adults** % (95% CI)
**Sensitivity**	95.13 (93.48–96.47)	94.93 (92.43–96.80)	95.34 (92.89–97.13)
**Specificity**	36.08 (28.60–44.09)	32.58 (32.02–43.34)	40.58 (28.91–53.08)
**PPV**	89.05 (87.84–90.15)	87.29 (85.58–88.82)	90.89 (89.13–92.39)
**NPV**	57.58 (48.62–66.06)	56.86 (44.31–68.60)	58.33 (45.56–70.08)

*CI* confidence interval, *PPV* positive predictive value, *NPV* negative predictive value

Univariable analysis comparing adult cases and paediatric cases found the odds ratio (OR) of predictive ability of intra-operative findings to be 0.781 (CI 0.574–1.063) in adults and 1.041 (CI 0.99–1.093) in paediatric cases (Table 3). However, these differences were not statistically significantly (*p* = 0.068). Similarly there was no significant difference between consultant operator (OR 1.172 [CI 0.788–1.744]) and trainee (OR 0.976 [0.921–1.034]) with a *p*-value of 0.251. A significant difference (*p* < 0.001) was seen when comparing the predictive power of intra-operative findings between males and female. Females were less likely to have an accurate diagnosis of acute appendicitis intra-operatively OR 0.521 (95% CI 0.385–0.706). Multivariable analysis also showed no significant difference between age and primary operator, but did show that sex of the patient was significant with OR 0.47 (95% CI 0.329–0.673, *p* = <0.001).

**Table 3:** Univariate and multivariate analysis for predictive histopathological diagnosis of acute appendicitis based on intra-operative findings (*n* = 1021)

		**Univariate analysis** OR (95% CI)	**Multivariate analysis** OR (95% CI)
**Age**	Adult	0.781 (0.574–1.063)	0.771 (0.538–1.106)
	Paediatric	1.041 (0.990–1.093)	*p* = 0.158
		*p* = 0.068	
**Sex**	Female	0.521 (0.385–0.706)	0.470 (0.329–0.673)
	Male	1.119 (1.058–1.184)	*p* < 0.001
		*p* < 0.001	
**Primary operator**	Consultant	1.172 (0.788–1.744)	1.215 (0.767–1.922)
	Trainee	0.976 (0.921–1.034)	*p* = 0.407
		*p* = 0.251	

*CI* confidence interval

## Discussion

Appendicitis is a common clinical condition encountered in general surgical practice, but it continues to pose diagnostic and management challenges as highlighted by the Right Iliac Fossa Pain Treatment (RIFT) Study Group [11]. It needs to be considered as a differential for anyone who presents with acute abdominal pain. Some of the long-standing factors that have been utilised in an attempt to pre-operatively diagnose appendicitis include measuring the serum C-reactive protein (CRP) and assessing for leucocytosis. In the vast majority of cases of acute appendicitis these are elevated and contribute to helping physicians in making a diagnosis. However, alone these parameters are not robust diagnostic measurements [12]. The absence of specific and sensitive biomarkers or hallmark clinical findings pushes the official diagnosis of appendicitis to the post-operative stage relying on histopathology. Risk scores, such as the Alvarado score, have been developed to provide more comprehensive scores, which do help increase diagnostic sensitivity, but lack specificity [13]. The appendicitis inflammatory response score is another scoring system that, when compared to the Alvarado score, has greater specificity and a higher positive predictive value [14], but still makes preoperative diagnosis difficult. Diagnostic accuracy is particularly challenging in female patients of child-bearing age [11].

Our findings have demonstrated that surgeons have a high level of sensitivity at identifying acute appendicitis on gross examination at the time of surgery. This is applicable to both trainees and consultant surgeons. These findings are supported by a number of studies that have demonstrated no significant difference in the standard of care between trainees and consultants for laparoscopic appendicectomies [15] and for emergency laparotomies [16]. This is encouraging as it suggests that the opportunity for trainees to operate in theatre does not lead to an inferior standard of care for patients and that safe, appropriately supervised surgical training remains the gold standard in current surgical training.

Studies on this topic have consistently found that female patients present with greater difficulty for accurate diagnosing and appropriate management [11, 17]. The most recent of these is the RIFT study, which demonstrated that females had higher false negative rates (19% vs 7.2% in males, *p* = 0.007) and males had higher false positive rates (43.3% vs 22.2% in females, *p* = 0.05) in diagnostic accuracy of appendicitis pre-operatively. Additionally, women were more than twice as likely to undergo a negative appendicectomy when compared to men (28.2% vs 12.1%, respectively) [11]. Our findings have demonstrated similar patterns with being female having a significant risk of inaccurate intra-operative diagnosis of acute appendicitis. The preoperative discordant diagnosis of appendicitis in women is easier to explain with the broader range of differentials compared to men and the potential absence of specialist gynaecological input prior to surgery to narrow the diagnosis. However, this does not explain why intra-operative diagnosis for women is still relatively inaccurate.

In agreement with our findings, surgeons appear to be able to make more accurate diagnosis of acute appendicitis with lower false positive rates, but false negative rates remain higher [7, 15]. Diagnostic laparoscopy still remains a reliable investigation for suspected appendicitis. Application of the LAPP score has been shown to provide excellent diagnostic ability with a PPV of 99% and NPV of 100% [17]. Additionally, early laparoscopy in female patients with an ambiguous diagnosis could provide them with a faster diagnosis and discharge from hospital when compared to observation alone and reduce rates of negative appendicectomy [18, 19]. Jones et al. recommended a set vocabulary of normal, ‘inflamed’ or ‘gangrenous or perforated’ for surgeons to describe intra-operative findings, whilst any doubt of diagnosis should be classed as normal [9]. They suggested that this would improve diagnostic accuracy and van den Broek et al. have demonstrated that it can be safe to leave an appendix that is macroscopically normal with approximately 1% of patients returning with proven appendicitis and 91% going on to have no recurrent pain after 4 years [20]. Given the findings in current literature on the implications of removing normal appendixes, routine removal of a normal appendix should not be undertaken as it can have greater morbidity, mortality, longer hospital stays and increased costs for healthcare services [5, 21].

With ongoing controversy between leaving a normal appendix and the associated ‘avoidable’ morbidity associated with removing a normal appendix, it is crucial that robust pre-operative and intra-operative strategies exist to accurately identify patients with acute appendicitis. In this study we identified some discordance between histological evidence of acute appendicitis and intra-operative impression. Therefore, other clinical variables and not just macroscopic appearance alone should be used when deciding to perform appendicectomy.

## Data Availability

Available on request.
